# Radiological approaches to COVID-19 pneumonia

**DOI:** 10.3906/sag-2004-160

**Published:** 2020-04-21

**Authors:** Şule AKÇAY, Tevfik ÖZLÜ, Aydın YILMAZ

**Affiliations:** 1 Pulmonary Diseases Department, Faculty of Medicine, Başkent University, Ankara Turkey; 2 Pulmonary Diseases Department, Faculty of Medicine, Karadeniz Technical University, Trabzon Turkey; 3 Atatürk Chest Diseases and Thoracic Surgery Centre, Health Sciences University, Ankara Turkey

**Keywords:** COVID-19, pneumonia, chest computerized tomography

## Abstract

COVID-19 pneumonia has high mortality rates. The symptoms are undiagnostic, the results of viral nucleic acid detection method (PCR) can delay, so that chest computerized tomography is often key diagnostic test in patients with possible COVID-19 pneumonia. In this review, we discussed the main radiological findings of this infection.

## 1. Introduction

COVID-19 infection has been spreading globally. By 2 April 2020, there were 950,430 confirmed cases with COVID-19 infection in 203 countries [1]. COVID-19 is highly infectious and may lead severe pneumonia. The most common presenting clinical symptoms are fever and cough in addition to other nonspecific symptoms including dyspnoea, headache, muscle soreness, and fatigue [2]. 

Due to the symptoms and manifestations being nonspecific in COVID-19 pneumonia, special diagnostic tools have to be used. Viral nucleic acid detection using real-time polymerase chain reaction (RT-PCR) remains the standard test in the diagnosis of COVID-19 pneumonia. However, waiting-period for viral detection with RT-PCR can cause delayed diagnosis of pneumonia. Meanwhile, PT-PCR could result negative at the beginning of COVID-19 pneumonia. Sometimes, the interval between initial negative to positive RT-PCR can be 4–8 days [3,4]. Therefore, thoracic radiology evaluation is often key diagnostic element in patients with suspected COVID-19 pneumonia [4].

In this article, a wide spectrum from the preparation before the radiological test to the radiological findings on possible or confirmed COVID-19 cases are going to be discussed.

## 2. Preparedness 

The radiology department has an obligation to ensure patient-centred care while maintaining staff safety. Because radiology departments have the potential of being in the eye of spreading infection, plans to prevent nosocomial infection spread should be developed. The health authority of the authors (Ministry of Health of Turkey) has developed a pandemic preparedness checklist for medical clinics that can be applied to radiology clinics [5].

Preventing patient-to-staff transmission:

It is advised to use barrier protections in reception and ensure appropriate distance between patients and staff. Personal protective equipment (PPE) for staff involved in direct patient care of potential COVID-19 cases should include gloves, surgical masks (airborne precautions including N95 masks are not indicated), eyeglasses, and gowns. 

Preventing patient-to-patient transmission: 

It is strongly recommended to supply patients with hand sanitizer and surgical masks at entrances. It is an obligation to separate the patients with respiratory symptoms from those without symptoms.

Preventing staff/facility-to-patient transmission: 

Hand sanitizer can be handed out the staff. It is also an important point to ensure that, appropriate disinfection methods are used on clinical equipment including radiography equipment between uses, and the staff with respiratory symptoms should be off. Effective communication between the referring services and radiology department is crucial on limiting the spread of this highly contagious infection. 

The radiology department should be notified before transferring the suspected case so that appropriate infection control measures are in place beforehand. After imaging the suspected patient, the imaging room should not be used for 15 min [5]. 

## 3. Radiological findings and classification

In literature, the diagnostic value of chest x-ray is relatively low as 30–60% in COVID-19 pneumonia [6]. Although it is possible to see some abnormalities in viral pneumonia on chest x-rays, it cannot be excluded the disease if the chest x-ray is normal. Noncontrast chest CT should be considered for early diagnosis of viral disease in suspected patients with normal chest x-ray [7,8].

Some typical radiological images can be demonstrated by computerized tomography (CT) in COVID pneumonia patients [9]. The predominant CT findings of COVID-19 infection are bilateral, peripheral, and basal predominant ground-glass opacity, consolidation, or both.

**Figure 1:**
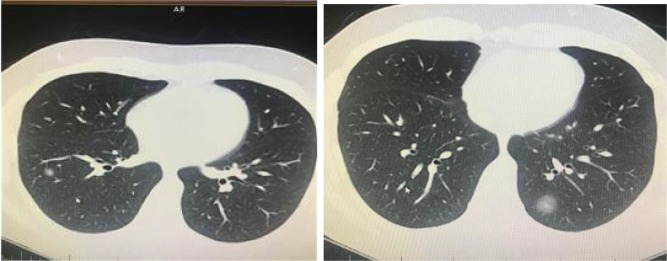
A 32-year-old man was admitted to our clinic with symptoms of fever and cough. PCR was positive for novel coronavirus. Focal small patchy infiltrate (atypical appearance) was seen in right and left lower lobe (archives of Şule Akçay).

**Figure 2:**
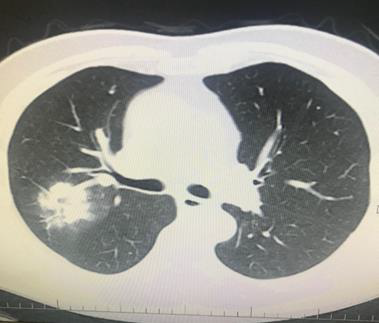
A 66-year-old man’s chest CT imaging, PCR positive, and CT has right upper lobe consolidation which is atypical appearance for COVID-19 pneumonia (archives of Şule Akçay).

**Figure 3:**
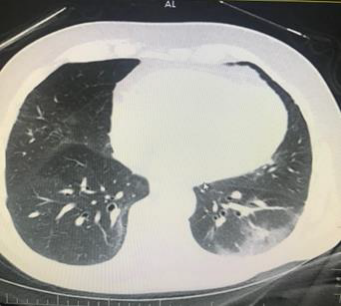
A 66-year-old man with chronic renal failure. PCR was negative. However, his wife has diagnosed as having COVID-19 pneumonia by PCR positivity. Ground-glass opacity was seen on left lower lobe, and treatment of COVID-19 pneumonia was applied (archives of Şule Akçay).

**Figure 4:**
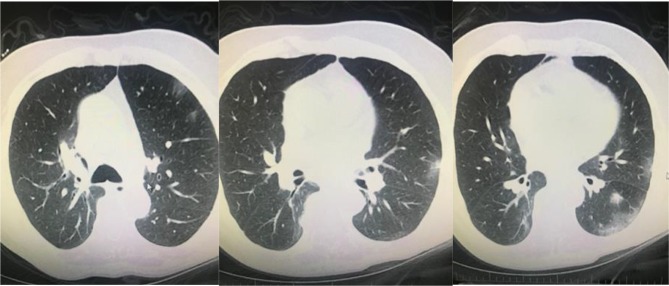
A 47-year-old man with chronic renal failure. PCR was positive. Chest CT revealed bilateral peripheral patchy infiltration (archives of Şule Akçay).

**Figure 5:**
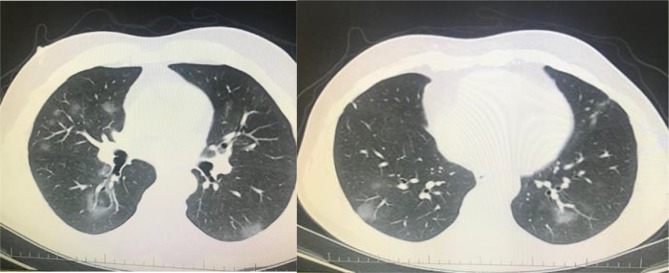
A 62-year-old man, PCR positive. Bilateral multilobar GGO on his chest CT (archives of Şule Akçay).

**Figure 6:**
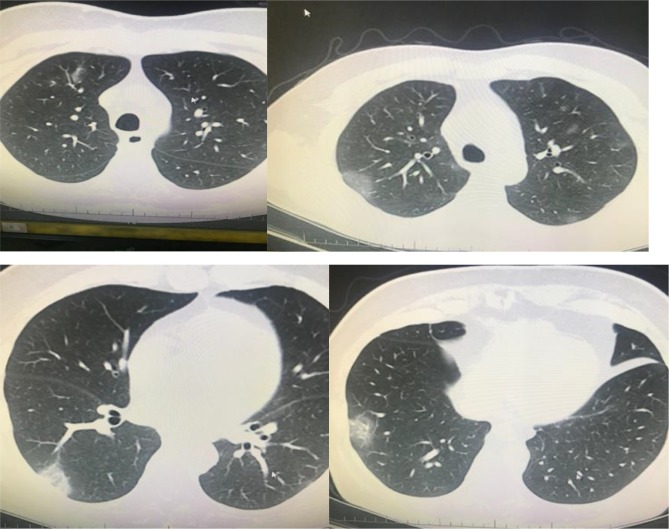
A 61-year-old man, PCR positive, unilateral multilobar focal consolidation areas on his chest CT (archives of Şule Akçay).

**Figure 7:**
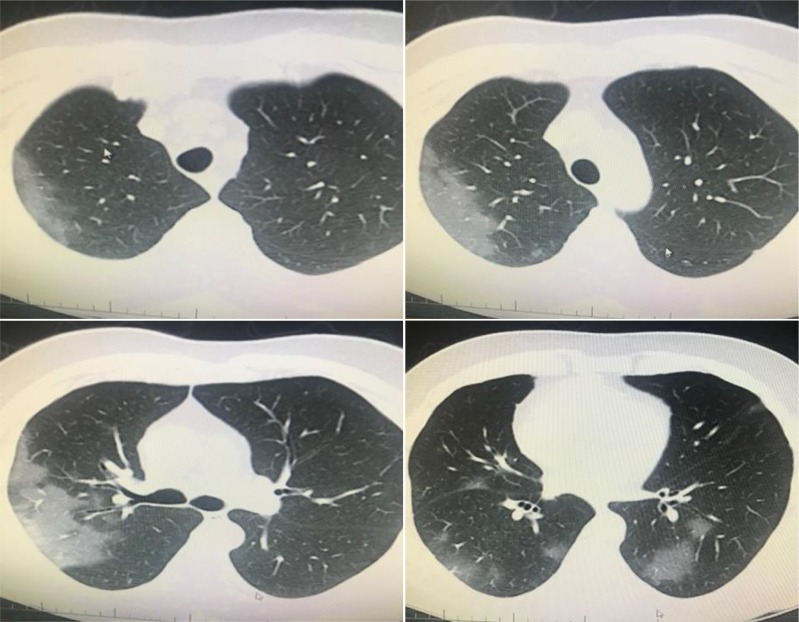
A 72-year-old man, PCR was negative. However, his wife’s swab was PCR positive. His chest CT has GGO in both lungs’ multilobes as typical COVID-19 pneumonia, early pneumonia treatment was essential according this imaging (archives of Şule Akçay).

**Figure 8:**
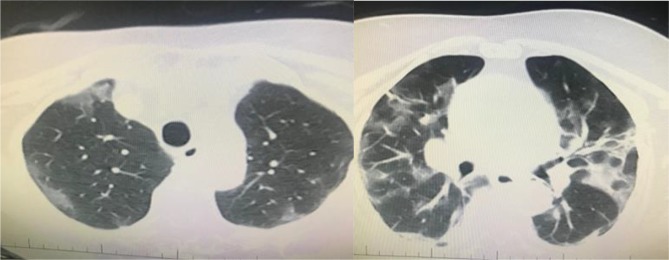
A 55-year-old woman, PCR was positive, bilateral multilobar GGO was seen in her chest CT (archives of Şule Akçay).

There are many similar articles about CT findings on pandemic of COVID-19 infection which are written by experienced authors from China, Italy, UK, and Canada in the literature. The first confirmed COVID-19 case in Turkey was detected on 10th of March 2020. Our knowledge and experiences about COVID-19 have been rising from the day that the first case was declared. According to our own experience, the predominant CT findings in COVID-19 pneumonia included basically ground-glass opacification, consolidation, bilateral involvement, and peripheral and diffuse distribution (Figures 1–8, from archive of Şule Akçay who is a pulmonary specialist of a tertiary hospital, Başkent University).

With the gradual recognition of radiological findings of COVID-19 pneumonia, guidelines, and reviews are written for rapid and correct diagnosis and treatment [9].

Radiologists are trying to classify the findings of thoracic CT images in COVID-19 pneumonia. The developed chest CT table which is advised by British Society of Thoracic Imaging (BTSI) could make classifications at 4 stages. This classification has been adopted by many radiologists in Turkey (Table 1) [10,11].

**Table 1:** Chest CT findings according to structure by British Society of Thoracic Imaging in COVID-19 pneumonia.

Pattern	Appearance
Classical COVID-19 (100% compatible)	Peripherally, lower lobe predominant, multiple, bilateral* ground glass opacities (GGOs) +Crazy paving Peripherally consolidation** Air bronchogram Converse halo sign/perilobullary pattern**
Possible COVID-19 (71–99% compatible)	Peripherally, lower lobe predominant, bronchocentric consolidation Converse halo sign/perilobullary pattern** Limited GGOs
Indeterminate (<70% compatible)	Incompatible with the other 3 radiological groups. Compatible with radiological appearance but has another diagnosis such as ILD, CTD.
COVID-19 exclusion (<70% compatible with another diagnosis)	Lobar pneumonia Cavitation Tree in bud/nodularity Lymphadenopathy Pleural effusion Advanced pulmonary fibrosis

*>1 lesion can be unilaterally.

**Organised pneumonia pattern.

Another classification has also been recommended by partnership of Radiological Society of North America (RSNA), Society of Thoracic Radiology (STR), and American College of Radiology (ACR) (Table 2) [12,13]. 

**Table 2:** Expert consensus statement on reporting chest CT findings related to COVID-19 (endorsed by RSNA, ACR, and STR).

Classification	Rationale	CT Finding	Suggested reported language
Typical	Commonly reported imagingfeatures of greater for specifity for COVID-19 pneumonia	Peripheral, bilateral (multilobar) GGO*, consolidation, or visible intralobular lines	Commonly reported imaging features of COVID-19 pneumonia are present.
Indeterminate	Nonspecific imaging features of COVID-19 pneumonia	Multifocal, perihilar, unilateral GGO or nonrounded or nonperipheral	Imaging features can be seen with COVID-19 pneumonia
Atypical	Uncommonly or not reported features of COVID-19 pneumonia	Isolated lobar or segmental consolidation, discrete smallnodules, cavitation or interlobular septal thickening, pleural effusion	Imaging features are atypical or uncommonly reported for COVID-19 pneumonia, alternative diagnosis should be considered
Negative	No features of pneumonia	No CT features to suggest of pneumonia	No CT findings present to indicate pneumonia

*GGO: Ground-glass opacities.

COVID-19 pneumonia series were firstly reported by China. Based on a prospective analysis of 1014 patients, the sensitivity of CT is estimated to be 97% in detecting COVID-19 infection. The sensitivity of the RT-PCR has been reported to be in the range of 60% to 70% [14]. Although PCR is the gold standard for confirming infection, chest CT has been shown to be more sensitive for the detection of COVID-19. Polymerase chain reaction–negative cases with positive CT findings and high clinical suspicion may benefit from repeated PCR testing [15]. 

In another Chinese series, the authors emphasized that COVID-19 pneumonia manifested with chest CT imaging abnormalities, even in asymptomatic patients, with rapid evolution from focal unilateral to diffuse bilateral ground-glass opacities that progressed to or coexisted with consolidations within 1–3 weeks. Combining assessment of imaging features with clinical and laboratory findings could facilitate early diagnosis of COVID-19 pneumonia [16]. The sensitivity of the CT was 97.2%, while the sensitivity of initial rRT-PCR was 83.3%. Patients with typical CT findings but negative rRT-PCR results should be isolated [17].

In an Italian study from Rome, the value of chest CT imaging was analysed in patients with confirmed COVID-19 pneumonia. The typical pattern of COVID-19 pneumonia was peripherally ground-glass opacities with multilobe and posterior involvement, bilateral distribution, and subsegmental vessel enlargement (>3 mm). Chest CT sensitivity was high (97%) but with lower specificity (56%) [18].

## 4. Radiological monitoring of recovery

In our country, a national guideline was written in the light of the recent literature and COVID-19 pneumonia patients who recovered from COVID-19 pneumonia, 4 stages of lung involvement were defined on CT [19-22]: 

1. Early stage (0–4 days after onset of the initial symptom): In this stage, ground-glass opacities (GGO) was the main radiological demonstration distributed subpleurally in the lower lobes unilaterally or bilaterally. 

2. Progressive stage (5–8 days after the onset of the initial symptom): In this stage, the infection rapidly aggravated and extended to a bilateral multi-lobe distribution with diffuse GGO, crazy-paving pattern, and consolidation.

3. Peak stage (9–13 days after the onset of the initial symptom): In this stage, the involved area of the lungs slowly increased to the peak involvement and dense consolidation became more prevalent. Findings included diffuse GGO, crazy-paving pattern, consolidation, and residual parenchymal bands.

4. Absorption stage (≥14 days after the onset of the initial symptom): In this stage, the infection was controlled and the consolidation was gradually absorbed. No crazy-paving pattern was present anymore. However, in this process, extensive GGO could be observed as the demonstration of the consolidation absorption.

In conclusion, chest CT examination plays an important role in the initial diagnosis and follow-up examination of the novel coronavirus pneumonia. Large studies are needed to clarify the diagnostic value of initial radiological evaluation and serial measurements in COVID-19 pneumonia management. 

## Acknowledgments

The authors (Akcay, Özlü, and Yılmaz) are the members of COVID-19 Advisory Committee of Ministry of Health of Turkey.
